# Timing Matters: Cross‐Codal Integration of Bridging Event Information Happens While Viewing Picture Stories

**DOI:** 10.1111/cogs.70276

**Published:** 2026-10-30

**Authors:** Ayşe Candan Şimşek, Frank Papenmeier, Julia Henner, Irina R. Brich, Markus Huff

**Affiliations:** ^1^ Foundation for Media in Education Leibniz‐Institut für Wissensmedien Germany; ^2^ Department of Psychology Eberhard Karls Universität Tübingen Germany

**Keywords:** Bridging inferences, Cross‐codal integration, Picture stories, Narrative comprehension

## Abstract

Despite the prevalence of multi‐codal narratives—such as comics consisting of pictures and text—in everyday life, little is understood about how individuals integrate information across different codalities to form cohesive mental representations. In the present study, we explored when processing costs emerged during cross‐codal integration of bridging event information in multi‐codal narratives. Participants were presented with uni‐codal (i.e., consisting only of pictures or of text) and multi‐codal stories in which the bridging event information was presented in the other codality. We measured viewing times for the bridging event and end‐state panels to reflect the cognitive costs associated with integration. The results showed a significant interaction between codality switch and panel position. While the presence of a codality switch increased viewing times for both panel positions, there was a much larger increase for the bridging event panel than the end‐state panel. This result supports a hypothesis suggesting that during narrative understanding, information of a different codality is integrated into the mental representation of the narrative upon encountering it. Our results underscore the influence of codality on narrative integration, indicating that understanding may be affected by the format in which information is presented. Future models of event cognition should incorporate codality as a factor in narrative comprehension.

## 1 Introduction

Comics are visual narratives that mostly involve two types of information: picture and text. Those can either appear together on a single panel, or they can be presented separately in different panels. Take the picture story in Fig. 1 from the book *A Boy, a Dog, and a Frog* (Mayer, 1967). The first panel shows the boy and dog running toward a tree branch while a frog sits on a leaf in the pond. Imagine that the middle panel is left blank. You should form an inference based on the initial and last information to fill in the blanks, which is called a *bridging inference* (Fincher‐Kiefer & D'Agostino, 2004; Magliano, Larson, Higgs, & Loschky, 2016; Magliano, Kopp, Higgs, & Rapp, 2017). Based on the last panel, where you see that the boy and dog fell into the pond, you can deduce that the boy and the dog stumbled. Now imagine that the middle panel is not left blank but replaced with a text description of the missing picture content (see Fig. 1b). Previous research shows that this type of *cross‐codal integration* is possible but is more costly than *uni‐codal integration*. For example, Huff et al. (2020) demonstrated increased processing costs at the end‐state panel when bridging event information was presented in another codality. However, because viewing times for the bridging event panel itself were not analyzed, it remained unclear when these integration costs first arise. The present study addresses this issue by measuring viewing times for both the bridging event and end‐state panels. We question whether people integrate verbal and pictorial codes when they first encounter information presented in a different codality, or whether such processing costs emerge later during subsequent narrative comprehension.

**Fig. 1:**
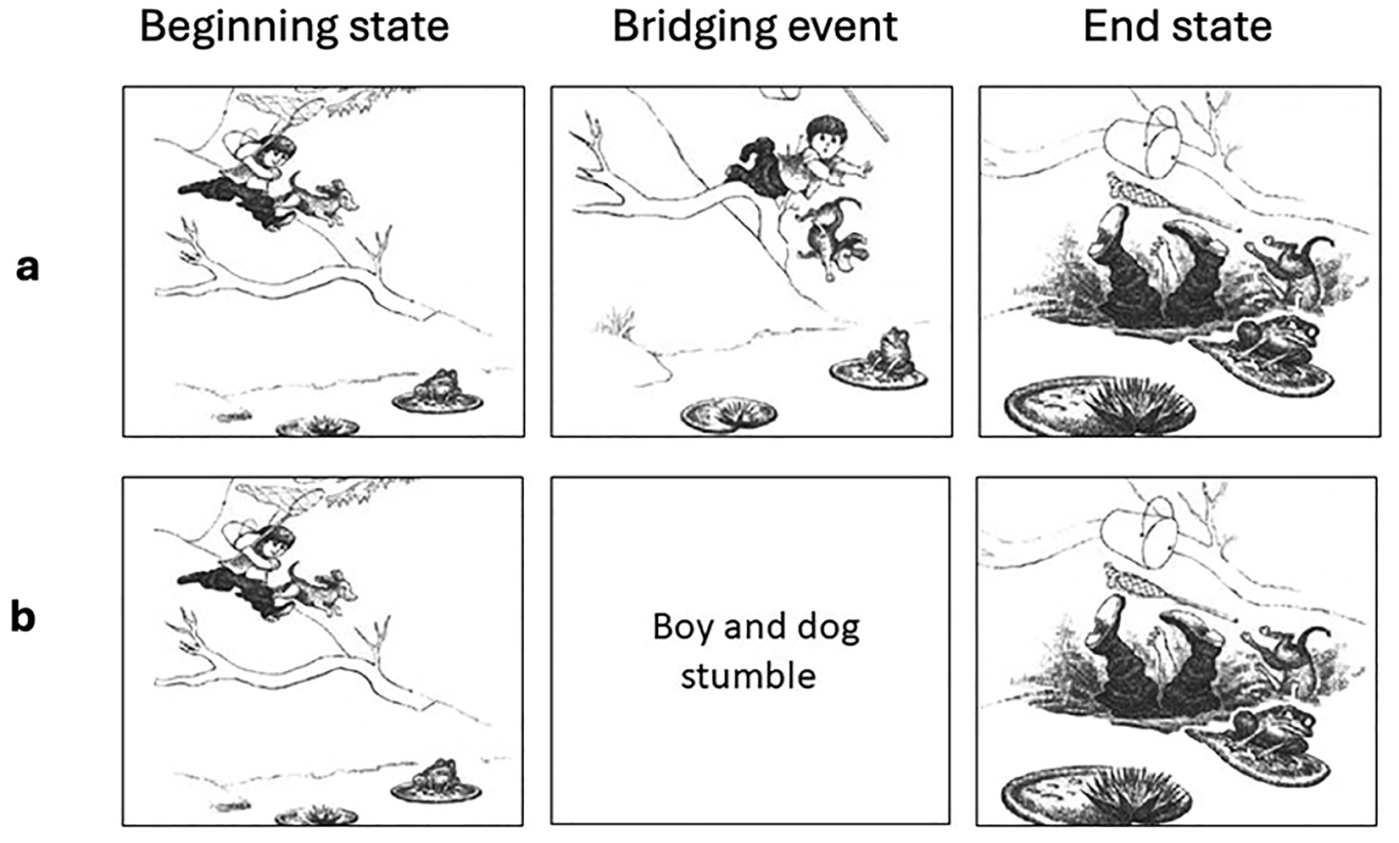
(a) Excerpt from the picture story “A Boy, a Dog, and a Frog” (Mayer, 1967). (b) Example of a cross‐codal bridging event within a picture‐based comic (Huff et al., 2020).

### 1.1 Event models in narrative comprehension

Contemporary research on event cognition can be linked back to early theories of language and narrative comprehension. One of the prominent theories in that area is the Structure Building Framework put forward by Gernsbacher (1990, 1997). According to Gernsbacher, a *general comprehension skill* is needed to understand a variety of narratives, either in written or visual form. This theory suggests that comprehension first requires the formation of a coherent mental representation, a “structure.” With every additional piece of information, this structure is either updated by mapping in new consistent information or by shifting to a new mental model when inconsistent information is encountered. The information that fits into the structure enhances the mental model, while any inconsistency leads to the suppression of the current structure in favor of a new one. Studies reported that people took longer to view the first sentence of a written narrative (Glanzer, Fischer, & Dorfman, 1984; Haberlandt, 1984) or picture of a visual narrative, compared to the following information (Cohn, 2014a; Cohn & Paczynski, 2013; Cohn & Wittenberg, 2015). Although first considered in relation to language comprehension, this theory also applies to visual narratives and inspired the event cognition theories.

Picture stories (i.e., comics) are a form of visual narrative. Studies have shown that people form coherent mental representations for picture stories (Cohn, 2013a). In addition, frameworks developed specifically for visual narratives, such as the Parallel Interfacing Narrative‐Semantics (PINS) Model (Cohn, 2020a), emphasize how narrative structure, meaning construction, and inference processes interact during comics comprehension. While early event cognition theories mostly focused on dynamic events, such as everyday activities and audiovisual narratives, as well as written narratives, recent theories such as the Scene Perception and Event Comprehension Theory (SPECT) have expanded this research to a variety of visual narratives, including comics (Loschky, Larson, Smith, & Magliano, 2020; Loschky et al., 2026). This theory suggests that event cognition relies on two types of processes: *front‐end* and *back‐end* processes. Front‐end processes are based on the salience of stimuli (shape, color, movement, etc.), which work to extract information during intake. Those depend on attentional resources and guide eye movements to the most crucial aspects of an event (Hutson, Smith, Magliano, & Loschky, 2017; Loschky, Larson, Magliano, & Smith, 2015). For example, eye‐tracking studies found longer viewing times for panels including text and pictures, compared to only pictures in picture stories (Chibaj, Tanaka, Shoji, & Toyama, 2007; Laubrock, Hohenstein, & Kümmerer, 2018). On the other hand, back‐end processes are based on prior knowledge and activate long‐term memory (Loschky, Hutson, Smith, Smith, & Magliano, 2018). Those are involved in the formation of the current event model and establishing a coherent mental representation based on time, space, and action. Back‐end processes are also related to the Structure Building Framework (Gernsbacher, 1990, 1997). While those lead to an initial construction of the foundation, any additional details that match the model are incorporated into this structure. If the information does not match the current structure, a shift to a new model is needed. Accordingly, each model is stored in episodic long‐term memory, although the previous model becomes less accessible when an event boundary is experienced.

Codality can be considered a medium‐specific feature within the context of SPECT. This theory already acknowledges that medium‐specific stimulus features can influence narrative processing, for example, through layout conventions, attentional guidance cues, panel sequencing rules, editing conventions, and the presence or absence of motion information (Loschky et al., 2020, 2026). These features can shape how viewers allocate attention and extract information from a narrative display, and motion information may influence the extent to which front‐end attentional selection is guided by back‐end event‐model knowledge (Hutson, Chandran, Magliano, Smith, & Loschky, 2022). Although much SPECT research has focused primarily on visual narratives, recent extensions of the Front‐end Back‐end Framework have begun to address modality and codality more explicitly (Magliano, Smith, Hu, Chandran, & Loschky, in press). The present study builds on this perspective by asking whether switching between textual and pictorial codalities within an unfolding narrative sequence creates measurable processing costs during comprehension.

At the front end, information must first be extracted from the currently presented stimulus. Textual and pictorial codalities differ at this stage because written words and depicted objects rely on partly distinct perceptual‐recognition processes. Written word recognition has been associated with left ventral occipitotemporal regions, including the visual word form area (Cohen et al., 2000), whereas object recognition has been associated with ventral visual regions involved in object and shape processing, including lateral occipital and occipitotemporal cortex (Kanwisher, Woods, Iacoboni, & Mazziotta, 1997). These differences provide a basis for treating text and pictures as codality‐specific at early stages of information extraction.

However, because the present study concerns integration of bridging event information into an unfolding narrative representation, codality‐switch costs should not be attributed solely to front‐end extraction. Integration also involves back‐end processes, including laying the foundation for an event model, mapping incoming information onto the current representation, updating causal and temporal coherence, and maintaining continuity across narrative segments (Gernsbacher, 1990, 1997; Loschky et al., 2018, 2020, 2026). Thus, cross‐codal switch costs may reflect multiple interacting stages rather than a single front‐end encoding process.

### 1.2 Bridging inferences

Connecting a series of discrete but interconnected information forms the basis of narrative understanding (Cohn, 2013a; Cohn, Paczynski, Jackendoff, Holcomb, & Kuperberg, 2012; Laubrock et al., 2018). In narratives of any form, a *bridging inference* is a deduction made to connect two points of a story. More crucial to this study, a *bridging event* is the information connecting two points of the same narrative. Research on bridging inferences shows that working memory is engaged in inferring missing parts, which puts a burden on cognitive resources (Fincher‐Kiefer & D'Agostino, 2004; Graesser, Singer, & Trabasso, 1994; Magliano et al., 2017). Bridging inferences require attention and mental effort, especially when the bridging event information is absent. The viewer needs to engage in forming links that would result in a coherent mental model of a visual narrative. Literature provides evidence for bridging inferences mostly in written narratives (Allbritton, 2004; Clark, 1977; Graesser et al., 1994; Haviland & Clark, 1974; Kintsch, 1988, 1998; Magliano & Graesser, 1991). However, this process also applies to picture stories. In a study on bridging inferences, Magliano et al. (2016) used the picture stories of the *Boy, Dog, Frog series* (Mayer, 1967, 1969, 1973, 1973, 1974; Mayer & Mayer, 1971, 1975; see Fig. 1a) where bridging event panel was either present or absent and collected viewing times during comprehension. People spent more time examining an end‐state panel in a picture story when the bridging event panel was absent (Magliano et al., 2016). Relatedly, Magliano et al. (2016) reported that both verbal and spatial working‐memory loads disrupted processing when bridging inferences were required in Boy, Dog, Frog picture stories. This finding is important because the materials were pictorial, yet both linguistic and visuospatial load manipulations affected processing. Thus, bridging inference generation may recruit multiple representational resources rather than relying exclusively on a single codality‐specific system. This provides converging evidence that event‐model construction may involve multi‐ or supra‐codal processes during narrative comprehension (Magliano et al., 2016). Also, Magliano et al. (2017) showed that people produced more false alarms for inferred bridging event panels but not for end‐state panels, suggesting that forming inferences leads to memory distortions. Other studies considered the type of information affecting bridging inferences. For example, Cohn and Wittenberg (2015) found longer viewing times for end‐state panels following action stars, suggesting that those necessitated the formation of bridging inferences. The question then arises as to how bridging inferences relate to distinct types of codes (i.e., text vs. picture) used to convey the narrative.

### 1.3 Bridging across different codalities

Two concepts are central to picture stories: the modality and codality of information. While there are conflicting descriptions in the literature (Clark & Salomon, 1986; Manfredi, Cohn, & Kutas, 2017), Huff et al. (2020) make the following distinction between them. As the sensory channel used to process a signal refers to the modality of the information (visual vs. auditory), the type of notation used to code the information (verbal, pictorial, symbolic) refers to the codality of the information. Accordingly, a picture with concurrent text (i.e., picture stories) can be categorized as unimodal as both information is in visual modality, while it can also be categorized as multi‐codal as it includes both verbal and pictorial codes. Theories such as the Structure Building Framework (Gernsbacher, 1990,1997), Event Indexing (EI; Zwaan, Langston, & Graesser, 1995), and the Event Segmentation Theory (EST; Zacks, Speer, Swallow, Braver, & Reynolds, 2007) argue that mental representations of events should be constructed similarly across codalities. Those theories, however, did not focus on what happens when information is distributed across different codalities within the same narrative. Verbal and nonverbal information differ in multiple aspects. Dual‐coding approaches distinguish between verbal‐symbolic and depictive representational formats (Paivio, 1991). Importantly, this distinction concerns coding format rather than complexity, as visual narratives such as comics can also involve structured conventions that guide comprehension (Cohn, 2013b). This difference can also be associated with how much implicit or explicit information is present in picture and text stories. While a text provides more explicit information using words describing emotions and intentions (Magliano, Loschky, Clinton, & Larson, 2013), a picture conveys more implicit information (Magliano, Kopp, McNerney, Radvansky, & Zacks, 2012; Magliano et al., 2013), where gestures and facial features are observed to derive the underlying internal state. Studies showed that both codalities also activate different areas of the brain (Ganis et al., 1996; Magliano et al., 2013).

Although the present study did not include neural measures, cognitive neuroscience research suggests that event comprehension across different input formats may recruit partially overlapping higher‐level systems. Such evidence is consistent with the possibility that codality‐specific front‐end extraction processes feed into more shared event representations during comprehension (Speer, Reynolds, Swallow, & Zacks, 2009; Yarkoni, Speer, & Zacks, 2008). We therefore treat codality switch as a process that may involve both format‐specific front‐end recognition and more general back‐end event model updating. Therefore, investigating how different codalities are processed and integrated during visual narrative comprehension becomes crucial.

One open theoretical question is when initially codality‐linked representations become more abstract or shared across formats during comprehension. Although perceptual simulation provides one possible mechanism through which verbal information could be recoded into a more depictive format, this does not imply that the resulting event representation is necessarily visual. Event representations may instead be multimodal, amodal, or distributed across multiple representational systems.

Only a few studies considered cross‐codal integration of text and picture information using comics. In one such study, Manfredi et al. (2017) used several types of text (descriptive vs. onomatopoeic) as bridging event information in picture stories. People reacted more to descriptive words, which suggested that they perceived those as more unexpected. In a more recent study, Huff et al. (2020) investigated cross‐codal integration of bridging event information by comparing uni‐codal and multi‐codal narratives. In picture stories, bridging event information was either in picture or text form, or it was absent or replaced by a blank panel. When the bridging event information was absent or in text form, the viewing times were longer, compared to when this information was in picture form. When the bridging event information was left blank, however, this led to the longest viewing times. Across three experiments, the authors showed that people can integrate information across codalities, but this process requires more cognitive effort than uni‐codal integration. As this study only examined end‐state panels but not bridging event panels, research is still needed to understand the relative timing of cross‐codal integration.

Prior work has examined processing at manipulated narrative segments and at subsequent segments in both visual and multimodal narratives, making these studies highly relevant to the present research question. For example, Zhang (2024) used four‐segment pictorial and textual stories with and without codality switch and compared viewing times across all segments of the narratives, allowing examination of how codality changes affected both the switched segment itself and later story processing. In visual narrative research, Cohn and Wittenberg (2015) used self‐paced viewing to investigate inferential processing following structural manipulations in comics, comparing processing at the manipulated segment and downstream segments. Similarly, Klomberg and Cohn(2022) examined several inferential techniques in visual narratives, including textual versus pictorial substitutions, and compared viewing times for the manipulated segment as well as the immediately following segment. Together, these studies show that self‐paced viewing methods can reveal how local narrative manipulations influence both immediate processing and subsequent comprehension. The present study extends this literature by directly contrasting immediate versus delayed integration accounts in codality‐switching narratives, focusing specifically on whether processing costs emerge at the bridging event panel itself or are deferred to the subsequent end‐state panel.

There may be several reasons for the costs associated with cross‐codal integration. *Recoding interference* can be one such cause (Adaval & Wyer, 2004; Huff & Schwan, 2008; Schooler & Engstler‐Schooler, 1990). Recoding interference refers to the cognitive cost associated with converting one codality into another. While a story using one codality (picture or text) first establishes a codality‐dependent representation, this mental model needs to integrate a second codality, which would be costly due to recoding interference. Huff et al. (2020) referred to these representations as *codality‐specific bases*, representing which codality (text‐base or picture‐base) is used to first establish the foundation of the mental model. While this is consistent in uni‐codal narratives, in multi‐codal narratives, *code‐switching* is necessary. Due to this difficulty, *perceptual simulation* (Barsalou, 1999, 2010; Louwerse, 2008; Zwaan, 2014) would be a likely scenario. According to perceptual simulation, verbal information is perceptually/visually recoded so that one does not need to represent two codalities simultaneously. This process bears similarities to the notion that mental representations of events are experienced similarly across codalities. Importantly, several prior comparisons of picture and text narratives used versions of the same Boy, Dog, Frog materials employed in the present study (Gernsbacher et al., 1990; Magliano et al., 2012). This increases the direct relevance of those findings for the current design because similarities or differences across codalities were examined using highly comparable narrative content. As people comprehend different uni‐codal narratives in the same manner (Gernsbacher et al., 1990; Magliano et al., 2012), event cognition studies also show similar segmentation regardless of codality (Baggett, 1979; Magliano et al., 2012). Those theories, however, did not focus on what happens when both codalities are presented in the same narrative. So, the question is whether the integration happens at the time of input, *immediate integration* (while people are reading or viewing a second codality), or later, *late integration* (when people are switching back to the first codality).

### 1.4 Experimental overview

This study investigates the relative timing of cross‐codal integration of bridging event information. To assess whether integration happens immediately or is delayed, we conducted an experiment in which people were exposed to 24 story clips of six panels. Those were part of six picture stories used previously by Magliano et al. (2016). Each story clip included a target episode depicting a beginning state, a bridging event, and an end‐state. The clips were either uni‐codal (picture‐only or text‐only) or multi‐codal (text and picture together) containing a cross‐codal bridging event panel. We measured the viewing times for the bridging event panel and the end‐state panel to assess the cognitive effort needed for integrating the bridging event into the narrative. Consequently, we inquired whether *codality switch* (yes vs. no) and *panel position* (bridging event vs. end‐state) affected viewing times.

There are two likely scenarios for locating the panel at which costs emerge when cross‐codal integration happens in narratives. On the one hand, the *immediate integration hypothesis* asserts that cross‐codal bridging event information is integrated into a narrative when encountered. Recoding interference suggests that additional cognitive resources are needed to integrate the cross‐codal information into the codality of an existing narrative. If this cost‐intensive codality switch occurs upon encounter, it should lead to longer viewing times when participants first encounter the cross‐codal bridging event information. Therefore, we expect an interaction between codality switch and panel position, indicating that while the presence of a codality switch would increase viewing times for both panel positions, there would be a much larger increase for the bridging event panel than the end‐state panel. This suggests that integrating information across codalities requires additional cognitive effort during the processing of the bridging event panel due to recoding interference. According to the *delayed integration hypothesis*, on the other hand, integration of the cross‐codal bridging event information does not happen immediately but at a later point in time upon realizing that this information is needed for comprehension. People then complement the current representation with the cross‐codal bridging event information after the fact. This hypothesis, therefore, assumes that the cognitive effort associated with cross‐codal integration is exerted not immediately after encountering the bridging event information but afterward when people are exposed to subsequent information. Consequently, we expect that while the presence of a codality switch would increase viewing times for both panel positions, there would be a much larger increase for the end‐state event panel than the bridging event panel. Overall, the delayed integration hypothesis suggests that integration and recoding interference happen at the point where the end‐state panel is encountered. Thus, the immediate and delayed integration accounts were treated as competing hypotheses, and the observed pattern of viewing times was expected to discriminate between them.

An alternative possibility is that some processing costs may become most evident after the bridging event panel. Under this delayed or downstream‐processing account, readers may begin processing the switched bridging event information when it appears, but additional updating, spillover, or coherence‐building demands may continue into subsequent narrative segments. In this case, increased viewing times would be expected at the end‐state panel, especially if readers need to relate the earlier cross‐codal bridging event to the later outcome of the story. This account is consistent with the use of end‐state viewing times in prior work on bridging inference processing and cross‐codal integration (Huff et al., 2020; Magliano et al., 2016), while allowing that initial switch costs may also arise earlier.

### 1.5 Open practices statement

We report how we determined our sample, all data exclusions, all measures, research questions, and analytic plans in the study. The experiment was approved by the local ethics committee of the Leibniz‐Institut für Wissensmedien, Tübingen, Germany. The participant data and analyses can be found at the OSF link: https://osf.io/f6q8d/.

## 2 Method

### 2.1 Participants

An a priori power analysis employing a critical power of 1 – β = 0.9 was conducted using the R package *powerbydesign* (Papenmeier, 2018). The required correlations, means and standard deviations were taken from the data analyses carried out by Huff et al. (2020). The power analysis revealed that a sample size of 85 participants was required to achieve a power of 0.9 for the predicted 2 × 2 interactions for a repeated‐measures analysis of variance (ANOVA), which was chosen as a rough approximation to gauge sample size based on our experimental design (especially for our within‐subjects design). Because the experiment used four counterbalancing lists, the final sample size needed to be a multiple of four to ensure equal allocation across lists. According to this power analysis, 88 participants (17 male, 70 female, one non‐binary) were recruited to participate in the experiment. Participants were recruited in a German university context and completed the German‐language materials. All participants reported sufficient German proficiency to complete the study.

Ethics approval was granted by Leibniz‐Institut für Wissensmedien, Tübingen, Germany. Recruitment was realized via a circular email sent to all members, students, and employees of Eberhard Karls Universität Tübingen, Germany. The participants were between 19 and 59 years (*M* = 27.29 years, *SD* = 8.58). Six participants did not provide information about their age. The broad adult age range was selected to obtain a heterogeneous adult sample while remaining comparable to prior narrative comprehension studies (Huff, Gagarina, Varkentin, & Brich, 2025).

### 2.2 Materials

The experimental stimuli used in this study are based on six picture stories from the *Boy, Dog, Frog* series illustrated by Mercer Mayer (Mayer, 1967, 1969, 1973, 1974; Mayer & Mayer, 1971, 1975) and the equivalent text versions created by Magliano et al. (2012). The picture versions of the stories were identical to those used by Magliano et al. (2016), with four additional pictures displaying only slight changes compared to their original picture. The English text versions were translated to German with the word length of bridging event and end‐state panels being adjusted to four to 12 words per sentence (*M*_BridgingEvent_ = 7.38 words, *M*_EndState_ = 7.46 words) based on Huff et al. (2020). Instead of employing the six stories, we presented participants with 24 individual clips taken from those stories. Each clip consisted of six panels and included one target episode. Target episodes comprised a beginning state, a bridging event, and an end state. The additional three panels were employed to give larger context to the target episodes, and we adopted the six‐panel clips from another recently published study (Brich, Papenmeier, Huff, & Merkt, 2024). The bridging events were identical to those validated by Magliano et al. (2016). Two uni‐codal and two multi‐codal presentation conditions were implemented in the experiment. In the uni‐codal picture‐only (PPP) and text‐only (TTT) conditions, the clips were presented in a uniform codality of either picture or text. The switch of codality was realized through the multi‐codal picture‐text (PTP) and text‐picture (TPT) conditions, which used a bridging event of the respective opposite codality as compared to the remaining clip. Fig. 2 depicts an example target episode of each presentation condition. Picture as well as text panels had a resolution of 1280 × 720 pixels and were presented full screen. The experiment was created using the program PsychoPy in version 2020.1.3 (Peirce et al., 2019).

**Fig. 2:**
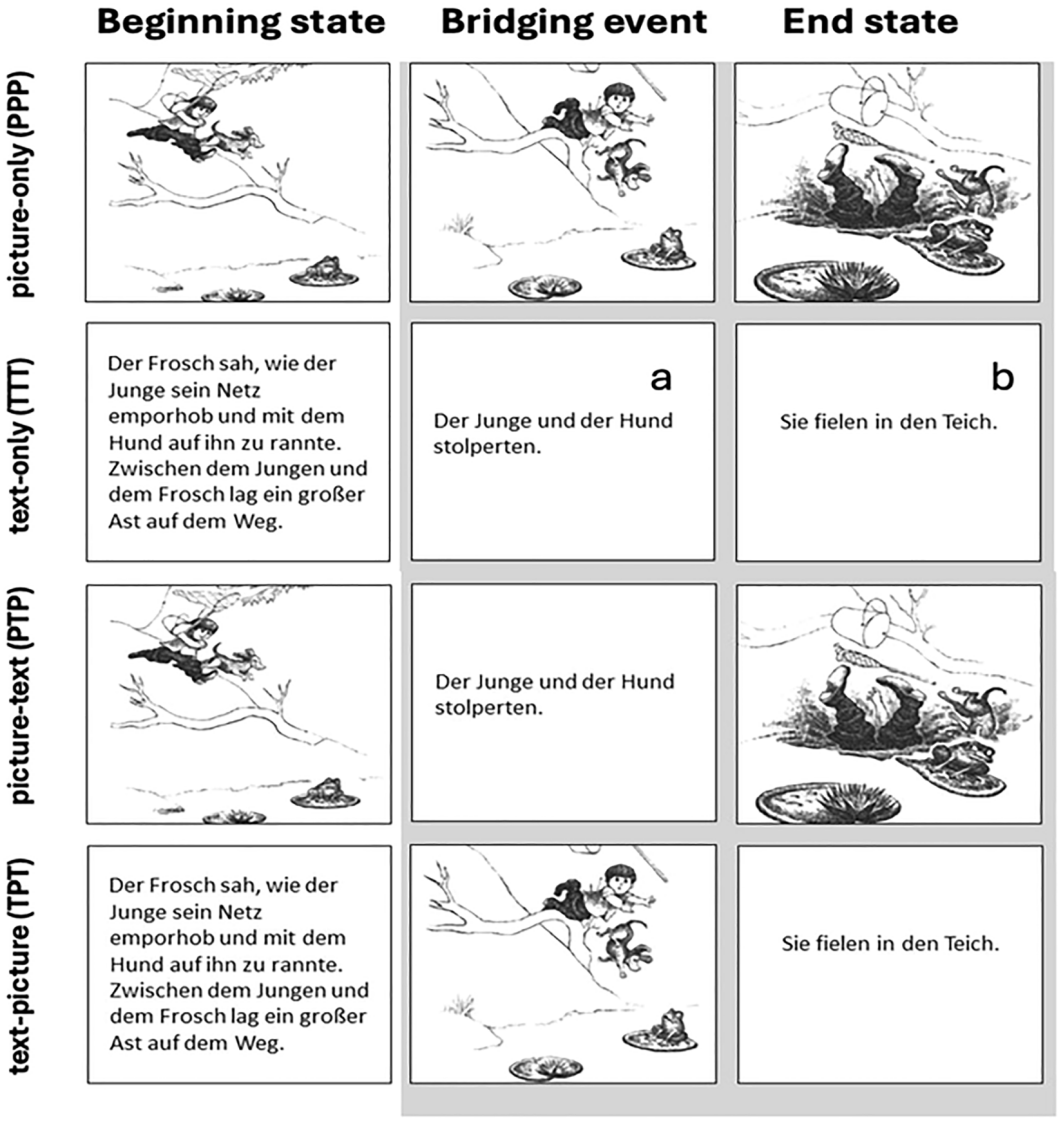
Example of a target episode across the four different presentation conditions. *Note*. See the English translation of the critical panels below. a. The boy and the dog stumbled. b. They fell into the pond.

### 2.3 Procedure

The study was conducted online using the Internet platform pavlovia.org. To take part in the experiment, participants received a link to the study. After providing informed consent via a designated keypress, participants were instructed that they would be presented with 24 short clips taken from different comic stories. They were informed that some clips would be presented comprising only text‐based information, some only picture‐based information, and that some would consist of a combination of textual and pictorial panels. Each panel of every comic clip was presented separately. By pressing the spacebar on their keyboard, participants were able to proceed to the subsequent panel in a self‐paced manner. This approach builds the foundation for employing the viewing time paradigm consistently used in the same manner in previous studies (Huff et al., 2020; Magliano et al., 2016). In the case of our study, the viewing time was defined as the time from the onset of the respective panel until pressing the space bar. Therefore, the viewing time indicates the amount of time spent viewing a single panel of a comic clip. Further, the instructions clarified that the participants should focus on comprehending the depicted plot. To reinforce this instruction and to maintain participants’ attention to the presented information, they answered two content questions at the end of each clip. These questions referred to basic story content (e.g., the protagonist or emotional reactions of characters) and served as comprehension checks only. The responses were therefore not analyzed. This procedure was broadly similar to Huff et al. (2020), although the previous study used summary‐style responses rather than yes/no questions. Participants were allowed to take short breaks between the presentations of the 24 individual clips. After all clips were presented, demographic data regarding age and gender were collected. At the end of the experiment, participants were able to enter a draw, giving them the opportunity to win a voucher worth 15 euros in return for their participation. The overall duration of the experiment was approximately 20 min.

### 2.4 Design

This study employed a within‐subjects design as each participant experienced every experimental condition. As the dependent variable, the viewing times spent on the critical panels (bridging event and end‐state panels) of the presented clips were measured. The independent variables were *codality switch* and *panel position*. This leads to a 2 (codality switch: yes vs. no) × 2 (panel position: bridging event vs. end state) design. The experiment used four different counterbalancing versions so that each clip would be presented in every presentation condition once. Each of the 88 participants was assigned to one counterbalancing version, which resulted in every clip being presented 22 times in each presentation condition. The order of the presented clips was randomized across participants.

## 3 Results

Data were analyzed using R (version 4.4.2). Prior to the analysis, the data were pre‐processed. The exclusion criteria regarding the viewing times were based on those established by Magliano et al. (2016). We first applied criterion‐based trimming, setting the critical panels' minimum and maximum acceptable viewing times to 480 ms and 20 s. If participants looked at a panel for less than 480 ms, it was unlikely that they could fully comprehend the information conveyed by the panel (Magliano et al., 2016). Therefore, we excluded all panel viewing times beneath this criterion from the analyses (95 of 4224 trials—2.25%). Applying the upper cut‐off of 20 s resulted in excluding four of 4224 panel viewing times (0.09%). In a following normative trimming, all panel viewing times that exceeded the arithmetical mean of each presentation condition by three standard deviations were excluded (76 of 4125 trials—1.84%). Eventually, if more than 25% of all trials of a single participant had to be excluded based on the criteria described above, the data of this respective participant were removed entirely from the analyses. This applied to two participants.

In the next step, the log‐transformed viewing time data were analyzed by means of a linear‐mixed‐effects model using the R package *lme4* (Bates et al., 2014). The model included the factors codality switch (yes vs. no), panel position (bridging event vs. end state), and their two‐way interaction as fixed effects. For the selection of the random‐effect structure, we followed the procedure proposed by Barr, Levy, Scheepers, and Tily (2013) and Scandola and Tidoni (2024) to reduce the risk of inflated Type 1 error of models that include only random intercepts for participant and item. That is, we started with a correlated and then uncorrelated maximal random‐effect structure. Because both models did not converge, we continued with a full complex random intercepts (CRI; Scandola & Tidoni, 2024) model, which was singular and thus reduced by removing both CRIs with zero variance (participant:position and participant:position:switch), which led to a nonsingular model. The model's parameters are presented in Table 1. We performed a Type‐II ANOVA with Satterthwaite degrees‐of‐freedom approximation.

**Table 1:** Model fit

	Log‐Transformed Viewing Time		
Predictors	Estimates	CI	*p*
*(Intercept)*	0.61	0.52–0.70	<.001
*switch [yes]*	0.17	0.11–0.23	<.001
*position [End State]*	−0.01	−0.09–0.06	.756
*switch [yes] × position [End State]*	−0.10	−0.17–−0.03	.008
*Random Effects*	
*σ^2^*	0.19
*τ_00 participant:switch_*	0.00
*τ_00 clip:position:switch_*	0.00
*τ_00 participant_*	0.08
*τ_00 clip:switch_*	0.00
*τ_00 clip:position_*	0.01
*τ_00 clip_*	0.01
*ICC*	0.36
*N _participant_*	86
*N _switch_*	2
*N _clip_*	24
*N _position_*	2
*Observations*	4033
*Marginal R^2^ / Conditional R^2^*	0.018/0.372

The ANOVA provided a significant main effect of codality switch, *F*(1, 22.35) = 24.52, *p* < .001, η_p_^2^ = 0.52, and but no evidence for an effect of panel position, *F*(1, 23.02) = 3.28, *p* = .083, η_p_^2^ = 0.12. Most important, there was also a significant two‐way interaction of codality switch and panel position, *F*(1, 23.08) = 7.12, *p* = .014, η_p_^2^ = 0.24 (see Fig. 3). Thus, the presence of a codality switch increased viewing times for both panel positions, with a substantially larger increase for the bridging event panel than the end‐state panel.

**Fig. 3:**
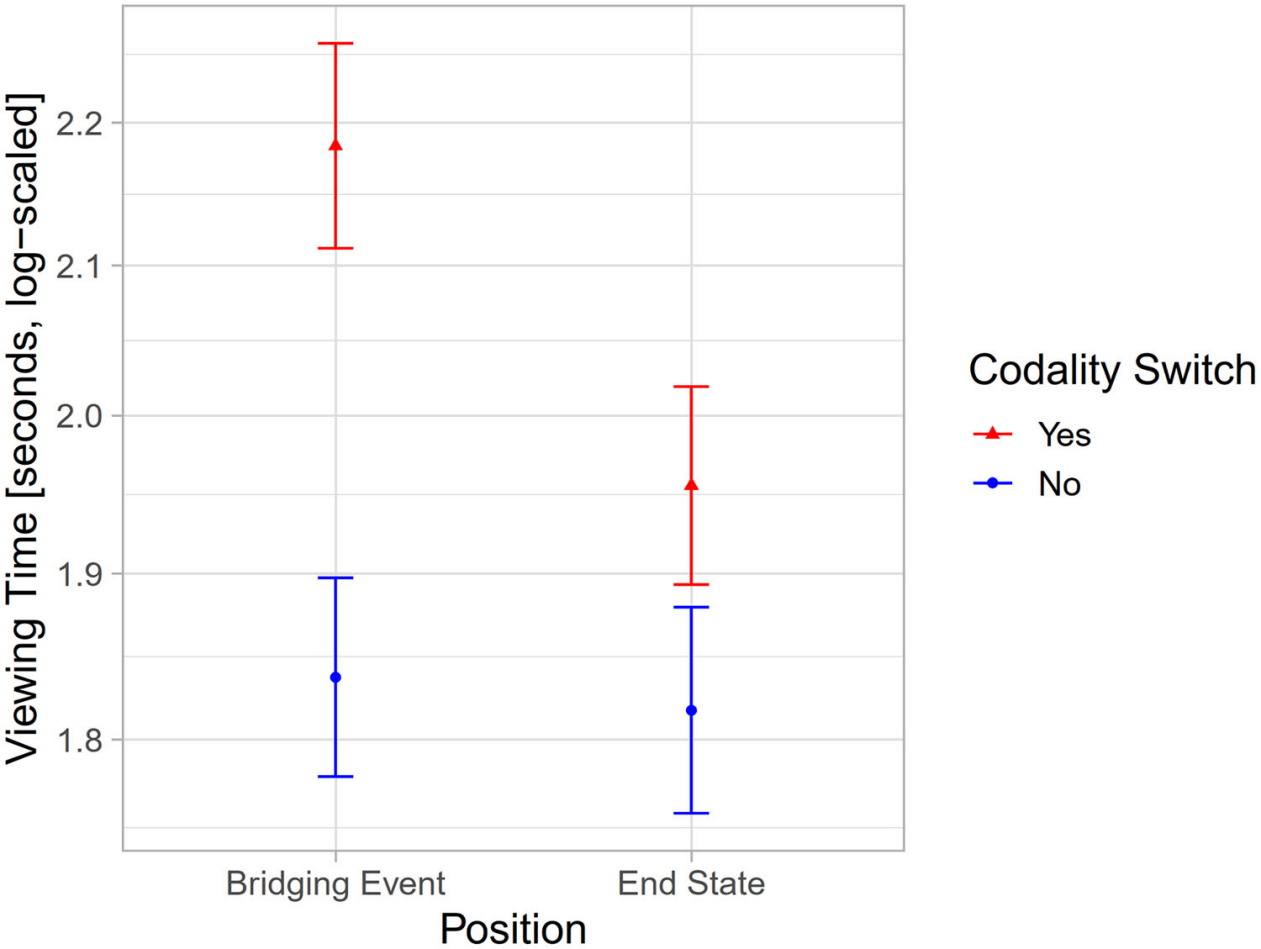
Viewing times as a function of panel position and codality switch. *Note*. Depicted are geometric means + SEMs. Raw reaction times were log‐transformed prior to aggregation. The y‐axis is log‐scaled but labeled with back‐transformed values in seconds for ease of interpretation. Standard Errors of the Mean (SEMs) were calculated on the participant level within the log‐scale before back‐transformation.

Using the emmeans package in R (Searle, Speed, & Milliken, 1980), we conducted a post hoc analysis to further investigate the significant interaction between panel position (bridging event vs. end state) and codality switch (with vs. without a codality switch) from our primary analysis. Estimated marginal means (EMMs) of codality switch were calculated within each level of panel position using the Satterthwaite method for degrees of freedom. This analysis confirmed that the presence of a codality switch significantly increased viewing times for both the bridging event, *t*(41.6) = −5.57, *p* < .001, *d*_z_ = −0.86, and the end state, *t*(41.4) = −2.40, *p* = .021, *d*_z_ = −0.37. Because the conditional *R* ^2^ of the model was lower than 0.6, we also computed paired comparisons on the aggregated data (see Scandola & Tidoni, 2024), which supported the EMM findings for both the bridging event *t*(85) = −9.43, *p* < .001, *d*_z_ = −1.02, and the end state, *t*(85) = −4.65, *p* < .001, *d*_z_ = −0.50. Thus, switching codalities from one panel to the next was associated with switching costs for both panels. However, these switching costs were much higher for the bridging event panel, where participants first encountered a codality switch, than for the end‐state panel, where codality was switched back to the base codality. This indicates that cross‐codal integration occurs right at the encounter of a new codality, supporting the immediate integration hypothesis.

### 3.1 Exploratory: Picture‐Text‐Picture (PTP) versus Text‐Picture‐Text (TPT)

This exploratory analysis examined whether codality‐switch costs differed as a function of switch direction: from text to picture within text‐based sequences (TPT) versus from picture to text within pictorial sequences (PTP). We investigated whether the codality switch by panel position interaction reported in our main analysis occurs equally for the integration of a text panel into picture stories and a picture panel into text stories or whether it might be mainly driven by one or the other. In order to directly compare the codality switch by panel position interaction between PTP and TPT without confounding this comparison by overall codality effects (such as participants viewing pictures faster than text or the other way around), we defined a new factor called comparison with the levels “Picture‐Text‐Picture vs. Control” and “Text‐Picture‐Text vs. Control” (see Fig. 4). For “Picture‐Text‐Picture vs. Control,” the PTP condition was compared against the TTT condition for the bridging event and compared against the PPP condition for the end state. For “Text‐Picture‐Text vs. Control,” the TPT condition was compared against the PPP condition for the bridging event and compared against the TTT condition for the end state. We analyzed the log‐transformed viewing time data with a linear‐mixed‐effects model using the R package *lme4* (Bates et al., 2014). The model included the factors codality switch (yes vs. no), panel position (bridging event vs. end state), comparison (“Picture‐Text‐Picture vs. Control” vs. “Text‐Picture‐Text vs. Control”), and all two‐way interactions as well as the three‐way interaction as fixed effects. For the selection of the random‐effect structure, we followed the same strategy as for our main analysis (see above), leading to a CRI model with random intercepts for participant, clip, participant:switch, participant:position:comparison, clip:switch, clip:position:switch, clip:position:comparison, and clip:position:switch:comparison. We performed a Type‐II ANOVA with Satterthwaite degrees‐of‐freedom approximation.

**Fig. 4:**
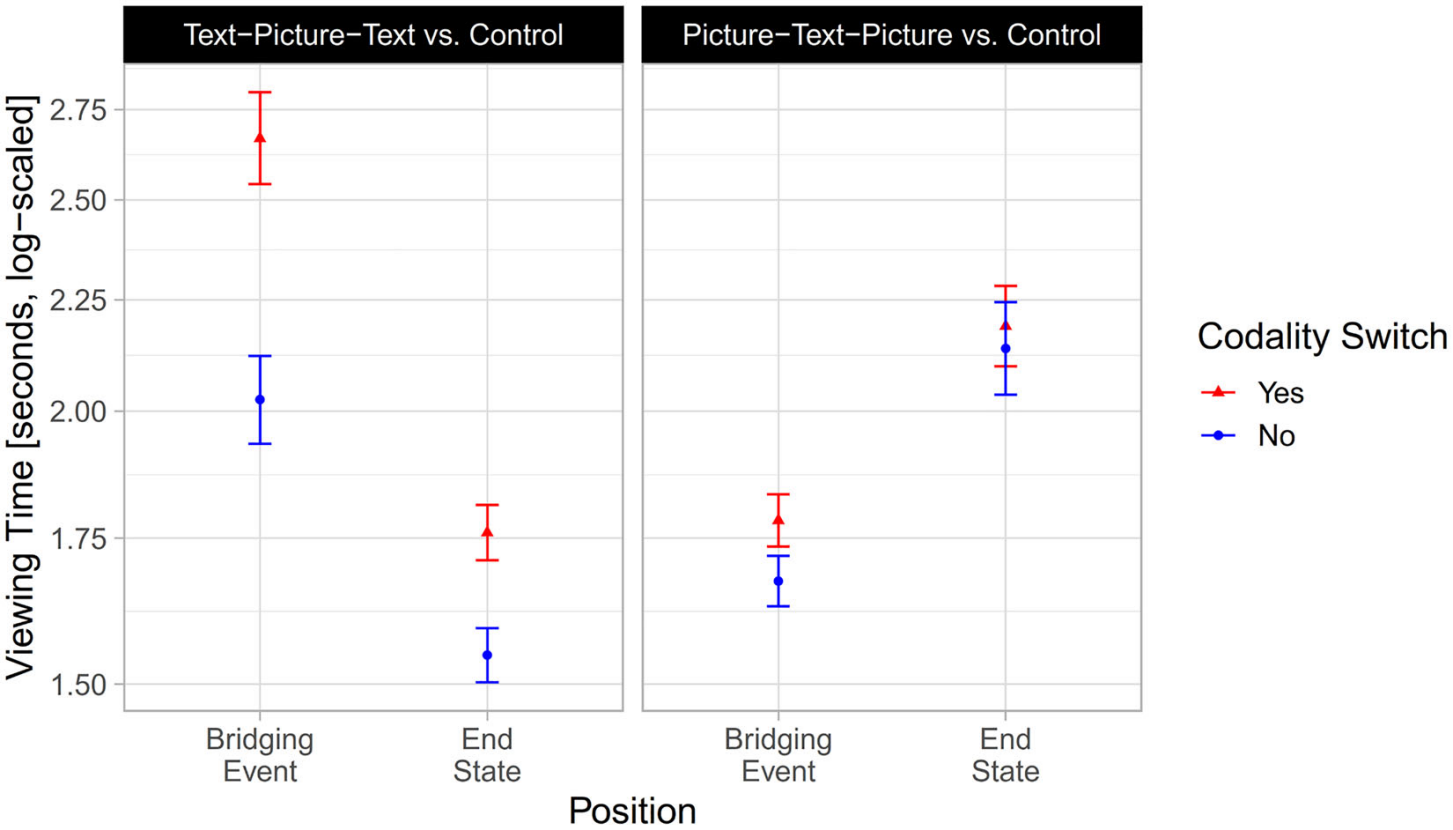
Viewing times as a function of panel position and codality switch, separated by whether codality switched to picture in text story or switched to text in picture story. *Note*. Depicted are geometric means + SEMs. Raw reaction times were log‐transformed prior to aggregation. The y‐axis is log‐scaled but labeled with back‐transformed values in seconds for ease of interpretation. SEMs were calculated on the participant level within the log‐scale before back‐transformation.

The ANOVA provided a significant main effect of codality switch, *F*(1, 27.34) = 25.07, *p* < .001, η_p_^2^ = 0.48. There was no evidence for a main effect of panel position, *F*(1, 89.58) = 2.49, *p* = .118, η_p_^2^ = 0.03, or comparison, *F*(1, 92.10) = 0.09, *p* = .766, η_p_^2^ < 0.01. There was a significant two‐way interaction of codality switch and panel position, *F*(1, 22.76) = 8.53, *p* = .008, η_p_^2^ = 0.27, a significant two‐way interaction of panel position and comparison, *F*(1, 92.10) = 51.21, *p* < .001, η_p_^2^ = 0.36, and a significant two‐way interaction of codality switch and comparison, *F*(1, 39.99) = 27.50, *p* < .001, η_p_^2^ = 0.41. There was no evidence for a three‐way interaction of codality switch, panel position, and comparison, *F*(1, 40.00) = 2.97, *p* = .093, η_p_^2^ = 0.07. That is, we again observed the interaction of codality switch and panel position with a stronger effect of codality switch for the bridging event than the end state. Importantly, we found no evidence for a three‐way interaction; thus, the results did not provide evidence that the pattern of stronger codality‐switch costs for the bridging event than the end state differed between TPT and PTP sequences. However, the significant codality switch by comparison interaction indicated that there was a much stronger overall codality switch effect for TPT than PTP, showing that the integration of a picture panel into a text story was associated with much higher integration costs than the integration of a text panel into a picture story. Note that the significant panel position by comparison interaction was not relevant for our main research question but simply reflects that the picture panels (bridging event panels for TPT vs. control and end‐state panels for PTP vs. control) were viewed longer than the text panels (end‐state panels for TPT vs. control and bridging event panels for PTP vs. control) in our experiment.

## 4 Discussion

This study investigated when processing costs emerged across narrative segments during cross‐codal integration in multi‐codal narratives, specifically focusing on picture stories that combine pictorial and textual information. We devised an experiment where we varied the base codality (text vs. picture), codality switch (yes vs. no), and panel position (bridging event vs. end panels). We have measured viewing times for bridging event and end‐state panels to reflect the mental effort exerted for integration (Clark, 1977; Cohn, 2014a; Graesser et al., 1994). Research on bridging inferences shows that when a critical information—a bridging event—is absent, people engage the working memory to infer the missing parts, which result in longer viewing times for an end‐state panel (Magliano et al., 2016, 2017). Other studies showed that when the bridging panel is present but in a different codality (i.e., when the bridging event is in text codality in a picture story), the viewing times for the end‐state picture were significantly longer in the text condition as compared to the picture condition, where every panel was in picture codality (Huff et al., 2020). As studies mostly concentrated on end‐state panels, investigation of bridging event panels is crucial to understand whether the integration happens early—immediate integration, at the time of encountering a bridging event of different codality, or it happens later—delayed integration, when people realize that the codality‐specific information is missing and the information coming from the other codality needs to be integrated into the mental representation.

The findings indicate that switching codality generally slowed processing, suggesting that integrating information across formats is more demanding than continuing within a single format. Importantly, these costs were substantially larger when participants first encountered the switched bridging event panel than when they subsequently viewed the end‐state panel. This suggests that additional processing demands arise when the new format must be incorporated into the developing narrative representation. Although some later updating may continue into subsequent panels, the strongest disruption occurred at the initial point of codality change. The larger increase in viewing times at the switched bridging event panel indicates that the strongest processing costs emerged when participants first encountered information presented in a new codality. Overall, this pattern is consistent with immediate integration demands, as readers may need to incorporate newly formatted information into the developing narrative representation at that point.

At the same time, local surprisal associated with the codality change may also have contributed to the increased viewing times (Huff et al., 2020; Pettijohn & Radvansky, 2016). Although smaller, the remaining switch cost at the end‐state panel suggests that processing consequences of the codality switch may continue beyond the initial encounter, potentially reflecting spillover, continued updating, or end‐of‐sequence wrap‐up processes. Also, the Multimodal Parallel Architecture framework characterizes the insertion of one codality into another as a form of substitution within an ongoing narrative structure (Cohn & Schilperoord, 2024). From this viewpoint, the increased viewing times observed at the switched bridging‐event panel may partly reflect the processing demands associated with integrating a substituted unit into a representational structure that had established expectations based on the preceding codality. Thus, the findings do not only imply that all integration is completed immediately; rather, they indicate that the strongest detectable processing costs occurred when the switched information first appeared.

The exploratory findings also highlight important differences from previous studies. Huff et al. (2020) reported codality‐switch costs in pictorial sequences, reflected in longer viewing times at the end‐state panel when the bridging event was presented in text. In contrast, the corresponding PTP end‐state effect appeared substantially weaker in the present data (see Fig. 4). Thus, although both studies suggest that switching between codalities can be cognitively demanding, the specific distribution of switch costs across narrative segments was not identical. One possibility is that differences in task demands, materials, or the inclusion of bridging‐event viewing times in the present study influenced where processing costs became most apparent. The present findings also differ from Zhang (2024), who reported switch effects for PTP but not TPT in an English‐speaking sample, whereas our exploratory analyses suggested a larger overall switch effect for TPT than PTP. Although these comparisons should be interpreted cautiously, they suggest that the magnitude and direction of codality‐switch effects may depend on characteristics of the materials, language background, familiarity with visual narratives, or other participant‐level factors. Future research designed specifically to compare these possibilities would help clarify the conditions under which different codality‐switch asymmetries emerge.

In addition, baseline differences between picture and text viewing times may partly reflect differences in visual complexity, information density, or the amount of detail available for inspection, in addition to codality‐related processing demands. In the present materials, several pictorial panels, particularly bridging event and end‐state panels, contain multiple visual elements that may require additional time to inspect and interpret. Accordingly, longer viewing times for pictures should not be attributed solely to codality. Interestingly, this pattern differs from Zhang (2024), who reported relatively longer processing times for textual materials under some conditions. Such discrepancies across studies may reflect differences in stimulus construction, sentence complexity, image detail, participant characteristics, language background, or procedural demands. Future work would benefit from more matching textual and pictorial stimuli for informational complexity and perceptual detail.

Also relatedly, the immediate costs observed following codality changes may pertain to broader switching phenomena described in task‐switching and bilingual code‐switching research, where transitions between representational systems typically produce processing costs at the point of switch (Monsell, 2003; Meuter & Allport, 1999). Research on bilingual language switching further suggests that such costs may vary depending on dominance, familiarity, and expectancy relations between systems. This perspective may help explain the asymmetry observed between TPT and PTP sequences in the present study. In addition, ERP studies of bilingual code‐switching have identified both early and later processing effects associated with language switches (e.g., Moreno et al.,^2002^ ), providing a potentially useful comparative framework for distinguishing front‐end and back‐end contributions to codality switching during narrative comprehension.

So, the present findings are broadly consistent with previous research suggesting that switching between codalities can impose measurable processing costs during narrative comprehension (Huff et al., 2020; Manfredi et al., 2017). In this sense, the results converge with earlier work showing that integrating information across representational formats is not cost‐free. At the same time, the specific directional pattern observed here does not fully replicate all prior findings. In particular, the stronger TPT than PTP effect in the present data differs from studies reporting larger switch costs in pictorial sequences, suggesting that the magnitude and direction of codality‐switch effects may depend on stimulus properties, participant characteristics, language background, familiarity with visual narratives, or procedural factors.

Our study also suggests that theories of narrative comprehension emphasizing shared or codality‐independent event representations may benefit from more explicit consideration of how representational format influences online processing. Frameworks such as the Structure Building Framework (Gernsbacher, 1990), the EI Model (Zwaan et al., 1995), EST (Zacks et al., 2007), and SPECT Theory (Loschky et al., 2020) have been highly influential in explaining how readers build coherent representations of events across contexts. The current results do not contradict these broader accounts but indicate that the format in which information is presented may shape the ease with which incoming information is processed and integrated during comprehension. At the same time, the present effects should not be interpreted as reflecting representational structure alone. Differences in perceptual complexity and baseline processing demands may also have contributed to the observed viewing‐time patterns. Thus, codality‐related effects may arise from an interaction between lower‐level perceptual processes and higher‐level integrative demands.

Overall, our findings concern local processing costs in experimentally controlled sequential presentations and should not be taken to imply that mixed‐codality narratives are inherently inefficient. In naturalistic comics reading, text and pictures often co‐occur predictably within a shared layout, and readers may rely on learned conventions for integrating the two formats. Moreover, cross‐codal facilitation is also possible: pictures may prime the processing of accompanying words and reduce rather than increase processing difficulty in familiar comics contexts (Rasamimanana, Mizzi, Melmi, Saffi, & Colé, 2025). Thus, the present effects are best understood as local costs arising from unexpected codality changes within controlled sequences rather than as a general property of all text‐picture combinations.

### 4.1 Limitations and future directions

One limitation of our study is that it was conducted online, which may introduce variability in viewing times due to non‐standardized data collection. Yet, as the data align with prior research, we are confident that our findings are robust. Our study focused exclusively on viewing times for the bridging event and end‐state panels. Examining viewing times across a wider range of panels, including the beginning state and subsequent panels, could offer a more comprehensive understanding of the cognitive processes involved in narrative comprehension. We also asked yes/no questions about the stories to control for comprehension and make sure that the participants attended to the panels. Future studies may combine brief comprehension checks with open‐ended recall measures, allowing researchers to assess both attention during the task and deeper narrative understanding after each story. Also, studies reported that people took longer to view the first sentence of a written narrative (Glanzer et al., 1984; Haberlandt, 1984) or picture of a visual narrative, compared to the following information (Cohn, 2014a; Cohn & Paczynski, 2013; Cohn & Wittenberg, 2015). Investigating whether this pattern holds in multi‐codal narratives would further elucidate the role of codality in narrative processing.

A further limitation is that we did not assess potentially relevant individual differences that may influence cross‐codal processing. For example, we did not measure participants’ prior experience with comics or other visual narratives, visual narrative fluency, or working‐memory capacity. Individual differences in visual language fluency may affect how efficiently readers interpret sequential images and integrate information across codalities (Cohn, 2020b), while working‐memory capacity may influence the maintenance and updating of narrative information during codality switch. These factors may therefore moderate the magnitude of codality‐switch costs observed in the present study.

Also, the longer viewing times observed for picture panels may partly reflect lower familiarity with interpreting sequential visual narratives relative to reading text. Future work should directly assess participants’ experience with comics and related visual media, for example using the Visual Language Fluency Index (Cohn, 2014b), and examine whether such individual differences interact with codality‐switch effects.

In addition, because self‐paced viewing times aggregate multiple processes across an entire panel, they provide coarser temporal resolution than eye‐tracking or electrophysiological methods. The present results therefore identify the panel at which processing costs were most evident rather than the precise fixation‐level or millisecond‐level timing of integration. Future work using eye‐tracking or Electroencephalography (EEG) could examine whether codality‐switch costs arise during early attentional orienting, front‐end information extraction, or later back‐end mapping and updating processes (Irwin, 1996; Kurby & Zacks, 2012). Also, eye‐tracking methods, which have previously been employed to compare processing of text and picture panels (Chiba et al., 2007; Laubrock et al., 2018), could help disentangle attentional orienting, surprisal responses, and integration‐related costs during codality switch. The consistency of the observed pattern across analyses suggests that the findings are reliable, although replication in future studies remains important.

As a related application, understanding the timing of cross‐codal integration has broader implications beyond narrative comprehension. In educational contexts, insights into how learners integrate textual and pictorial information can inform the design of instructional materials that facilitate learning. Multimedia learning theories suggest that simultaneous activation of both codalities in working memory enhances comprehension (Schnotz & Bannert, 2003). Our findings suggest that future research should examine whether codality effects observed here also generalize to applied educational contexts.

Finally, future research should explore how cross‐codal integration processes might differ in contemporary comics, which increasingly rely on pictorial elements over text (Cohn, Taylor, & Pederson, 2017). Comparing comics across different time periods could reveal how changes in codality usage affect narrative comprehension and integration processes. Such studies could deepen our understanding of the evolving dynamics between text and imagery in visual storytelling.

## 5 Conclusion

In conclusion, our findings provide greater support for the immediate integration hypothesis than the delayed integration hypothesis. Specifically, processing costs associated with cross‐codal integration in multi‐codal narratives were strongest when participants first encountered information in a different codality. This finding highlights the cognitive costs associated with integrating textual and pictorial information and suggests that narrative comprehension is codality‐dependent. Our results have important implications for theoretical models of event cognition and narrative comprehension, which should account for the role of codality in mental representation. Further research is needed to fully understand the mechanisms underlying cross‐codal integration and its applications in various domains, such as education and media design.

## Funding information

No funding was received for this study.

## Consent

All participants provided their informed consent before the experiment. All participants were made aware that this study was for scientific research. Additionally, all authors provided their consent for publication.

## Conflicts of Interest

The authors declare no conflicts of interest.

## Data Availability

The data and analyses for the experiment can be found at the OSF link: https://osf.io/f6q8d/.
